# Chemically synthesized Gb_3_ glycosphingolipids: tools to access their function in lipid membranes

**DOI:** 10.1007/s00249-020-01461-w

**Published:** 2020-09-19

**Authors:** Jeremias Sibold, Somayeh Ahadi, Daniel B. Werz, Claudia Steinem

**Affiliations:** 1 Georg-August-Universität Göttingen, Institute of Organic and Biomolecular Chemistry, Tammannstr. 2, 37077 Göttingen, Germany; 2 Technische Universität Braunschweig, Institute of Organic Chemistry, Hagenring 30, 38106 Braunschweig, Germany; 3 Max Planck Institute for Dynamics and Self Organization, Am Faßberg 17, 37077 Göttingen, Germany

**Keywords:** Fluorescence microscopy, Glycolipids, Model membranes, Organic synthesis, Shiga toxin

## Abstract

Gb_3_ glycosphingolipids are the specific receptors for bacterial Shiga toxin. Whereas the trisaccharidic head group of Gb_3_ defines the specificity of Shiga toxin binding, the lipophilic part composed of sphingosine and different fatty acids is suggested to determine its localization within membranes impacting membrane organisation and protein binding eventually leading to protein internalisation. While most studies use Gb_3_ extracts, chemical synthesis provides a unique tool to access different tailor-made Gb_3_ glycosphingolipids. In this review, strategies to synthesize these complex glycosphingolipids are presented. Special emphasis is put on the preparation of Gb_3_ molecules differing only in their fatty acid part (saturated, unsaturated, α-hydroxylated and both, unsaturated and α-hydroxylated). With these molecules in hand, it became possible to investigate the phase behaviour of liquid ordered/liquid disordered supported membranes doped with the Gb_3_ species by means of fluorescence and atomic force microscopy. The results clearly highlight the influence of the different fatty acids of the Gb_3_ sphingolipids on the phase behaviour and the binding properties of Shiga toxin B subunits, even though the membranes were only doped with 5 mol% of the receptor lipid. To obtain fluorescent Gb_3_ derivatives, either fatty acid labelled Gb_3_ molecules or head group labelled ones were synthesized. These molecules enabled us to address the question, where the Gb_3_ sphingolipids are localized prior protein binding by means of fluorescence microscopy on giant unilamellar vesicles. The results again demonstrate that the fatty acid of Gb_3_ plays a pivotal role for the overall membrane organisation.

## Introduction

### Glycosphingolipids

Glycosphingolipids (GSLs) are a subclass of glycolipids that are found in cell membranes of various organisms ranging from bacteria to humans. Being typically a minor component of the cell membrane, they are of utmost importance for biological functions that rely on lipid-lipid and lipid-protein interactions (Schnaar and Kinoshita 2015). The chemical structure of 90% of mammalian GSLs is based on glucosyl ceramide, whereas galactosyl ceramide serves as a precursor for the remainder. One can define several major GSL series based on their internal core carbohydrate structure. One can classify them into the ganglio (GalNAc-β-1–4-Gal), globo (Gal-α-1–4-Gal), lacto (Gal-β-1–3-GlcNAc-β-1–3-Gal), and neolacto (Gal-β1-4-GlcNAcβ-1–3-Gal) series. Based on the ganglio GSL series, gangliosides are synthesized, where sialic acids are linked to the glycan structure to produce negatively charged GSLs. In contrast, globosides are neutral lipid molecules.

For the analysis of GSLs, they are extracted from tissues and cells using organic solvents. Extraction procedures are optimized to precipitate and remove proteins and nucleic acids while maximizing solubilization of GSLs along with other lipids. The mixture is then subjected to thin layer chromatography (TLC) to monitor the purity and to separate different GSLs. After separation by TLC, 10^–12^–10^–9^ mol quantities of GSL can be chemically detected with orcinol, a reagent for hexoses and with resorcinol-HCl, a reagent for sialic acid. Of note, with these extraction procedures, GSLs are categorized by their glycan structure, whereas the lipid tails are not determined (Fahy et al. 2009).

It is known that GSLs interact with both intracellular as well as exogenous proteins and are critical for membrane organisation, signalling, and recognition events. For example, membrane GSL receptors are known for exogenous microbial virulence factors such as cholera toxin and vero toxins (Johannes and Römer 2010; Lingwood 2011). In this context, major effort has been put in developing synthetic strategies towards gangliosides and the interested reader is referred to an excellent review of Hunter et al. (Hunter et al. 2018). Here, we will solely focus on the less recognized globosides and in particular the globoside Gb_3_, which is the membrane receptor for vero toxins. Vero toxins, a family of *E. coli* produced AB_5_ toxins responsible for the pathology of the haemolytic uremic syndrome (HUS) (Johannes and Billet 2020; Karmali et al. 2010) as well as Shiga toxin are known to specifically bind to Gb_3_. Okuda et al. (Okuda et al. 2006) have shown that Gb_3_ synthase knockout mice are completely protected against vero toxin infection demonstrating the high specificity of these toxins towards Gb_3_.

### The globoside Gb_3_

**Fig. 1:**
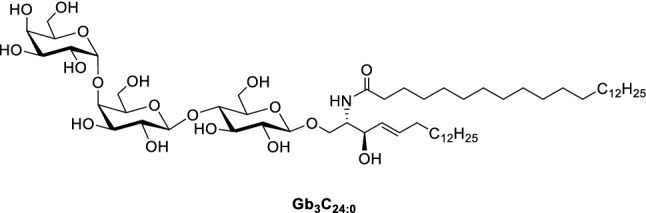
Structure of globotriaosyl ceramide (Gb_3_) with a saturated fatty acid (C_24:0_)

The globotriaosyl ceramide Gb_3_, also referred to as P^K^ blood group antigen and CD77 (Johannes and Römer 2010), is the major receptor lipid for a number of lectins. Just recently, Römer and co-workers have shown that Gb_3_ segregates into different domains at the plasma membrane of cells, which leads to a different binding behaviour of Shiga toxin compared to the lectin LecA from *Pseudomonas aeruginosa* (Schubert et al. 2020). The receptor lipid is composed of the trisaccharide α-D-galactose-(1 → 4)-β-D-galactose-(1 → 4)-β-D-glucose, bound via an acetal to a sphingosine moiety. One fatty acid is connected via an amide bond to the 2-amine of the sphingosine (Fig. 1).

While a number of studies investigated the specificity of protein binding to Gb_3_ (Johannes 2017), the variability of the ceramide backbone harbouring different fatty acids has been much less addressed. In various cell types [examples are (primary) human endothelial cell lines, human colon Caco-2, or HCT-8 epithelial cells (Kouzel et al. 2017) and references therein], a conserved repertoire of Gb_3_ species was found with saturated C_16:0_, C_22:0_, or C_24:0_ fatty acids and the unsaturated C_24:1_ fatty acid.

The ceramide lipid component of Gb_3_ has a major effect on Shiga toxin Gb_3_ binding (Kiarash et al. 1994; Pellizzari et al. 1992). The orientation of the carbohydrate moiety is dependent on both the lipid composition of the glycosphingolipid itself (Nyholm and Pascher 1993) and the membrane in which it is embedded (Lingwood 2011; Mahfoud et al. 2010; Watkins et al. 2014). Moreover, Lingwood and co-workers (Lingwood et al. 2010; Lingwood 2020; Mahfoud et al. 2009) suggested that the pathogenicity of Shiga toxin producing *E. coli* (STEC) infections is influenced by the different Gb_3_ species. Despite the importance of the fatty acid of Gb_3_, the majority of studies has been performed using Gb_3_ mixtures extracted from different sources, which vary in their fatty acid composition (Bien et al. 2020). Some early studies have determined the binding capacity and affinity of different Gb_3_ species towards Shiga toxin with the result that Gb_3_ sphingolipids with long fatty acid chains (C_20_-C_24_) have a higher affinity towards Shiga toxin than those with shorter chains (C_12_, C_14_) and that unsaturation and α-hydroxylation further enhance binding (Binnington et al. 2002; Kiarash et al. 1994). The finding that mixtures of Gb_3_ species behave differently than the sole components shows that the interaction with Shiga toxin is complex (Mahfoud et al. 2009). Even though such experiments were partially conducted in the presence of auxiliary lipids, results on membrane-embedded Gb_3_ species with a defined fatty acid are very rare. To be able to investigate the impact of Gb_3_ sphingolipids with respect to the chemical nature, synthetic strategies are required to obtain pure compounds. Moreover, to localize these Gb_3_ molecules in lipid membranes, fluorescent labels attached to Gb_3_ are highly desirable.

## Synthetic strategies to obtain Gb_3_ glycosphingolipids

Our ultimate goal was to synthesize fluorescent Gb_3_ glycosphingolipids that do not disturb the natural structure of a lipid bilayer and are still able to allow protein binding. Thus, we followed two directions:

*1) Introduction of the fluorophore as part of the fatty acid:* Numerous fluorescent fatty acids harboring NBD (Pagano 1990), BODIPY (Kaiser and London 1998), BOIMPY (Patalag et al. 2016), pyrene (Somerharju 2002) and other labels (Patalag and Werz 2016) have been developed in recent decades and have been successfully applied in various fields. However, the membrane organisation suffers either from their steric bulkiness and/or their most often polar properties. Therefore, we sought to integrate a non-bulky, non-polar fluorescent dye into the natural structure of the fatty acid. Building upon the work by Amat-Guerri and co-workers (Souto et al. 1994) and Thiele and co-workers (Kuerschner et al. 2005) we planned to introduce fluorescent conjugated pentaene and hexaene units as an integral part of the acyl chain.

*2) Attachment of a fluorophore to the carbohydrate head group:* To attach fluorophores to a hydrophilic carbohydrate head group has been demonstrated recently by Ando and co-workers and the dynamic behaviour of glycosphingolipids in membranes has been studied (Komura et al. 2016). Commonly the fluorophore is directly attached to the glycan part or via a linker. However, if the glycosphingolipid still needs to be capable of binding a specific protein, an exact knowledge of the protein in complex with the head group of the glycosphingolipid, as obtained by X-ray crystallography data, is required to identify the hydroxy groups of the sugars that are involved in binding and thus cannot be modified. If hydroxy groups are not involved and not completely buried in the binding site, they might be a point of attachment for a linker connected to a fluorophore or any other moiety (Isobe et al. 2007).

For both directions, modular chemical synthesis approaches were pursued that are described in the following chapters. Those readers, who are interested in the biophysical analysis of these synthetic molecules in the context of artificial lipid membranes, are directly referred to the chapters further back of this review.

### α-Hydroxylated nervonic acid

The first task to obtain the non-labelled and head group labelled Gb_3_ sphingolipids was to prepare the required fatty acids, either non-labelled also for the head group labelled Gb_3_ sphingolipids or fluorescently labelled. We decided on fatty acids with 24 carbon atoms as they are known to be a major part of natural Gb_3_ sphingolipid mixtures. Whereas saturated (C_24:0_) and unsaturated (C_24:1_) fatty acids are commercially available and configurationally defined and α-hydroxylated saturated derivatives had been synthesized in the past (Iwayama et al. 2009; Konen et al. 1975; Patterson et al. 1996), a synthetic route to α-hydroxylated unsaturated derivatives was not known. Oxidizing steps to introduce the α-hydroxy group as they are commonly employed in the synthesis of α-hydroxylated saturated congeners are not possible, because the C–C double bond would suffer.

**Scheme 1:**
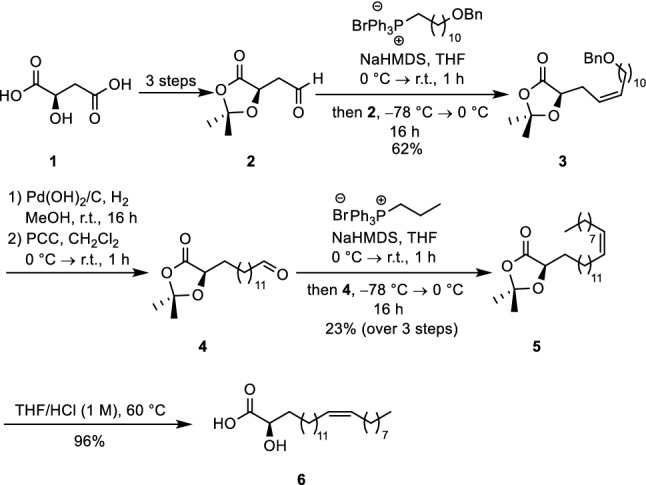
Synthesis of configurationally pure α-hydroxylated nervonic acid **6** from D-malic acid (Pawliczek et al. 2014)

Thus, we designed a novel synthetic route to α-hydroxylated nervonic acid using a chiral pool approach (Scheme 1). D-Malic acid (**1**) that contains a defined stereo centre was transformed by a literature-known protocol consisting of protection, reduction and oxidation into aldehyde **2**. In the next step, the carbon chain was elongated via a Wittig reaction using a phosphonium salt to furnish **3**. Hydrogenation of the olefinic moiety by Pd(OH)_2_ on charcoal under hydrogen atmosphere and removal of the benzylic protecting group, followed by subsequent oxidation with pyridinium chlorochromate (PCC) afforded aldehyde **4**. As the next step a (*Z*)-selective Wittig reaction was applied. Key is the use of a non-stabilized phosphorus ylide. Traces of the *trans*-stereoisomer could be easily separated by column chromatography. The isopropylidene protecting group was cleaved under acidic conditions affording target compound **6**. The respective fatty acid enantiomer that is found in bacteria was prepared in a completely analogous way starting from L-malic acid (Pawliczek et al. 2014).

### Fluorescent oligoene fatty acids

To access oligoene-containing compounds traditional approaches are Pd-catalysed reactions (either as cross-coupling reactions or as carbopalladation cascades of oligoyne units) or Wittig-type reactions. The latter are often superior for capricious compounds, because harsh reactions conditions such as high temperature can be avoided. Therefore, we designed a synthetic route to oligoene fatty acids based on Wittig-type reactions. Originally, Thiele and co-workers investigated this approach (Kuerschner et al. 2005). We modified the route so that it became shorter and the sensitive oligoene was introduced at a later stage by an elimination reaction.

**Scheme 2:**
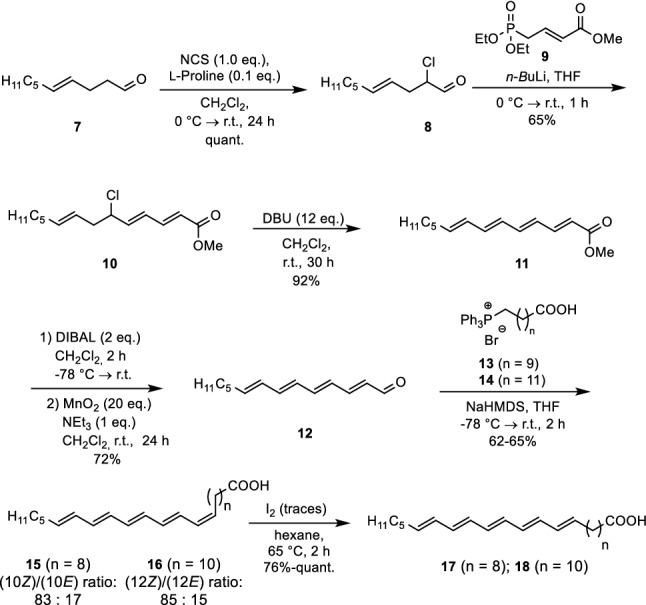
Synthesis of pentaene fatty acids **17** and **18** (Patalag and Werz 2012)

Starting point was commercially available (4*E*)-decenal (**7**) that was chlorinated quantitatively in α-position by *N*-chlorosuccinimide (NCS). The addition of L-proline acting as a proton shuttle was crucial. A Wittig-Horner reaction with phosphonate **9** afforded compound **10** in 65% yield. Problems with low-yielding Wittig-Horner reactions due to the conjugation were diminished by this procedure. After this coupling the elimination triggered by a large excess of DBU took place providing tetraene ester **11**. A reduction/oxidation procedure furnished aldehyde **12** as crucial intermediate for the next Wittig reaction with phosphonium salts **13** or **14** of different alkyl chain length. The low reaction temperature guaranteed a predominant formation of the (*Z*)-isomers **15** and **16** being non-natural counterparts to the common *cis*-configured unsaturated fatty acids. However, traces of iodine in boiling *n*-hexane catalysed an isomerization to the all-(*E*)-pentaene fatty acids **17** and **18** (Scheme 2). A similar synthetic protocol also secured the preparation of the corresponding fatty acids with six conjugated double bonds embedded into the carbon chain.

**Fig. 2:**
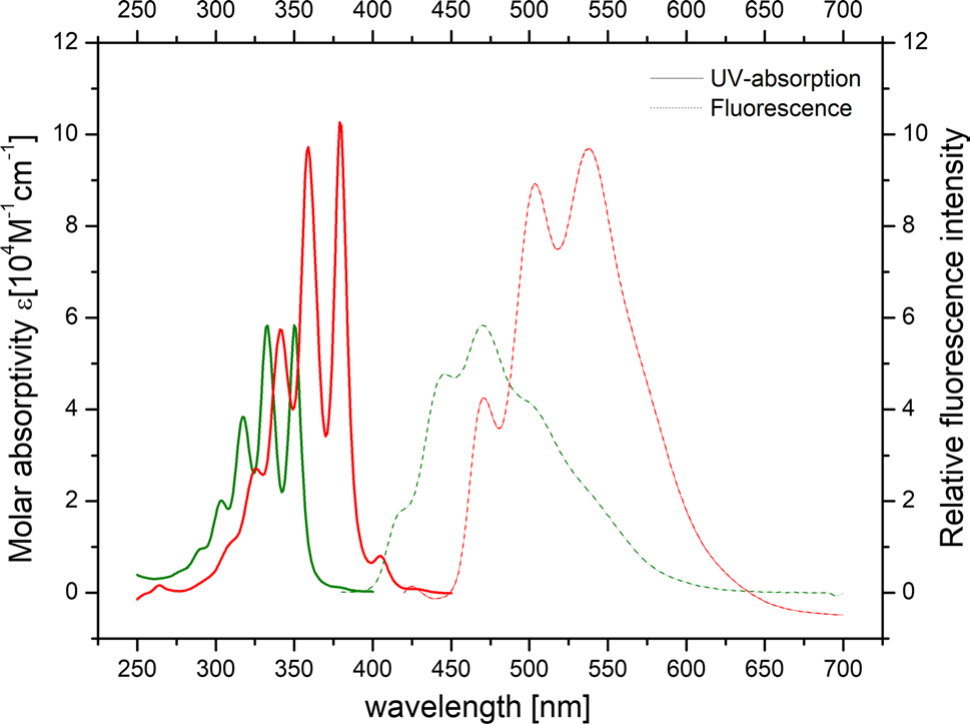
Excitation spectra (solid lines) and fluorescence emission spectra (dashed lines) of the all-(*E*) pentaene fatty acid (green) and the respective hexaene congener (red). Image taken with permission from (Patalag and Werz 2012)

Photophysical properties of these systems were investigated. Whereas the change of the proximal double bond from (*E*) to (*Z*) is with about 2 nm almost negligible, the difference between pentaene and hexaene fatty acids is striking (Fig. 2). Large Stokes shifts of 120 nm in pentaene and 159 nm in hexaene are observed (Patalag and Werz 2012).

The larger π-system shifts the UV-absorption maximum by about 30 nm from 350 to 379 nm to the red. Whereas the molar absorbance for the pentaene fatty acids are in the range of 60,000–70,000 M^1^ cm^−1^ (THF), these values increase for the hexaene fatty acids to 70,000–100,000 M^1^ cm^−1^ (THF). Although the slim shape and hydrophobic scaffold of the pentaene and hexaene fatty acids was expected to disturb the membrane integrity only mildly, these molecular entities turned out to be quite unstable if they were irradiated with laser light. Moreover, absorption far below 400 nm is not well-suited for fluorescence microscopes with a standard laser equipment. Hence, new fluorescent fatty acids with either a phenyl or a thienothienyl residue in conjugation to an oligoene system were designed with the idea to extend the π-system to red-shift the absorption and emission spectra and simultaneously increase the stability of the fluorescent core structure without increasing the bulkiness of the molecules too much.

**Scheme 3:**
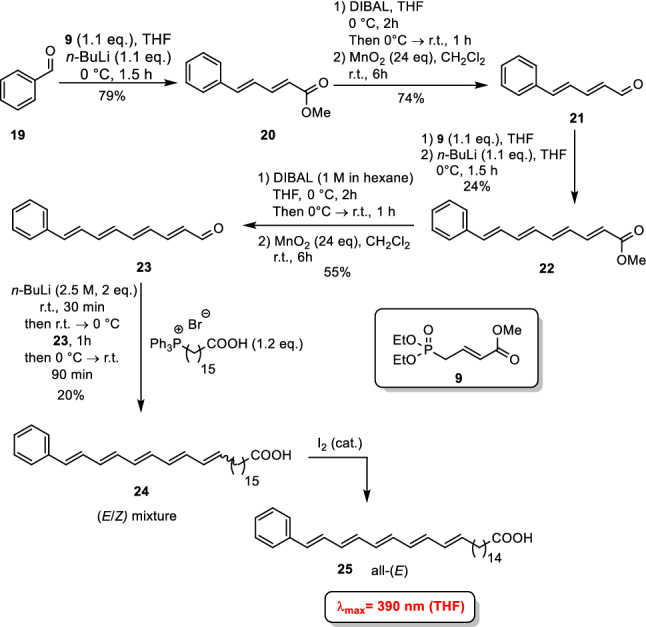
Synthesis of phenylpentaene fatty acid **25** (Patalag et al. 2017)

**Scheme 4:**
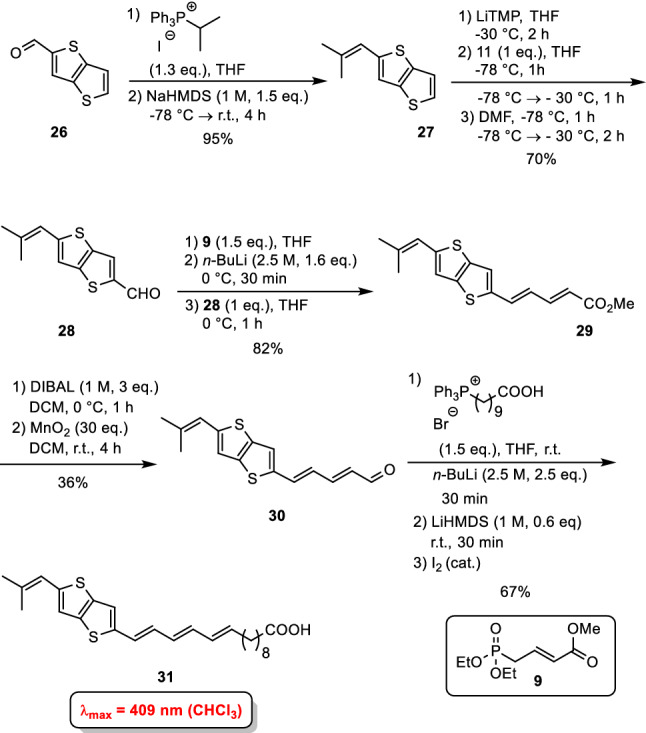
Synthesis of thienothienyl-modified fatty acid **31** (Patalag et al. 2017)

The synthesis is similar to fatty acids mentioned above using Wittig-type and reduction/oxidation procedures (Scheme 3). Starting material is benzaldehyde (**19**); the carbon chain gets successively elongated until compound **24** is reached. Finally, again an isomerization to the all-(*E*)-phenylpentaene had to take place. Due to the more extended π-system this compound absorbs at 390 nm and provides a higher photo-stability than the previously synthesized oligoene fatty acids. An even larger red shift was observed by embedding the very electron-rich thienothienyl moiety into an oligoene chain. The synthetic access to the thienothienyl-modified fatty acid **31** resembles the previously described ones (Scheme 4). First, thienothiophene-2-carbaldehyde (**26**) was engaged in a Wittig reaction to furnish isopropylidene-equipped compound **27**. A lithiation/carbonylation sequence using DMF as carbonyl source afforded aldehyde **28** which was subjected to a Wittig-Horner reaction using phosphonate. The emerging ester **29** was reduced and subsequently oxidized to aldehyde **30**. The latter was transformed by a Wittig reaction/isomerization procedure into the target compound. The photophysical investigations revealed an absorption maximum at *λ*_max_ = 409 nm. Also, this fatty acid proved to be much more stable than the oligoene fatty acids **17** and **18**. However, the drawback of these arene-substituted fatty acids is that (i) the fluorophore is not as slim as in the oligoene fatty acids and (ii) the π-system being stiffer than a saturated hydrocarbon chain is located at the terminus of the fatty acid and not in the middle of the hydrocarbon chain.

### Synthesis of non-fluorescent and fluorescent Gb_3_ glycosphingolipids with defined fatty acids

The power of chemical synthesis towards Gb_3_ glycosphingolipids is most promising because of the well-defined nature of the resulting material. Three major parts can be differentiated in a glycolipid which lead to the major retrosynthetic cuts: (i) the carbohydrate head group (hydrophilic part) and the ceramide (lipophilic part). The ceramide consists of a (ii) sphingosine such as d-erythro-sphingosine in mammalian glycolipids and (iii) a fatty acid which is linked to the amino group of the sphingosine.

**Scheme 5:**
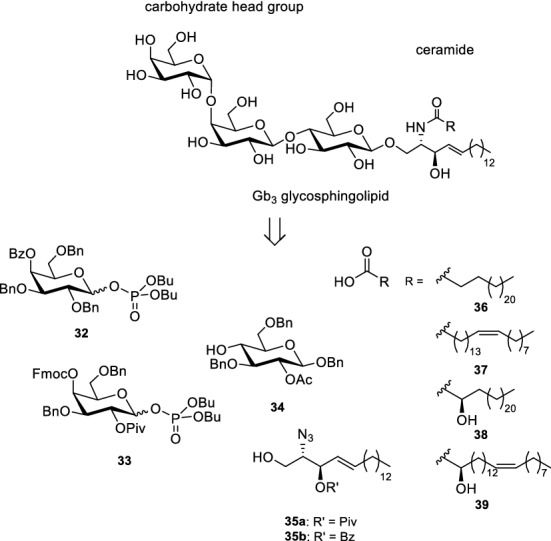
Retrosynthesis of Gb_3_ glycosphingolipids (Schütte et al. 2014, 2015)

**Scheme 6:**
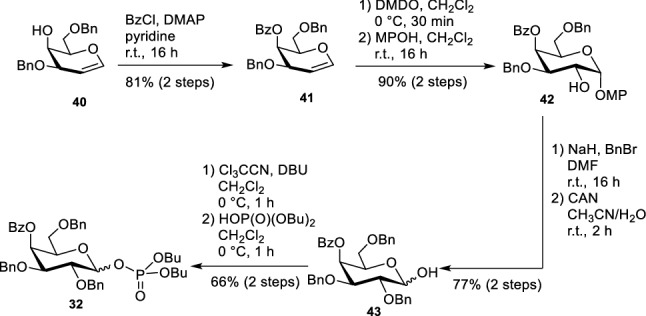
Synthesis of galactose building block **32** (Schütte et al. 2014)

In Gb_3_ the carbohydrate head group is a triaose which consists of two galactose moieties and one glucose. Whereas the sphingosine stays the same, the fatty acids vary in length, unsaturation and hydroxylation. Here, we concentrated our synthetic efforts on Gb_3_ molecules with 24 carbon atoms either saturated, mono-unsaturated, or α-hydroxylated. In addition, we also synthesized a Gb_3_ in which the fatty acid part is α-hydroxylated and unsaturated. The globotriaoside is traced back to three different monosaccharidic building blocks (Scheme 5, 32–34). Glucose derivative **34** was obtained in five steps starting from d-glucal that can be prepared in 100 g scale from D-glucose. The galactosyl building block **32** was synthesized starting from d-galactal (Werz et al. 2007). The third unit, an α-linked galactosyl acceptor, was obtained from 3,6-di-*O*-benzyl-D-galactal (**40**) (Scheme 6). A remote-participating group (Bz) was installed at the 4-hydroxyl group to facilitate the formation of the α-galactosidic linkage in the trisaccharide. DMDO-mediated epoxidation of galactal **41** followed by the attack of the emerging acetalic epoxide using *p*-methoxyphenol (MPOH) led to afforded acetal **42** in 90% yield. Benzyl ether formation and removal of the anomeric protecting group under oxidative conditions led to hemiacetal **43**. With trichloroacetonitrile under the basic catalysis the trichloroacetimidate was formed. Because sometimes phosphate donors are superior in glycosylation reactions (Ahadi et al. 2020) the respective trichloroacetimidate was converted into phosphate **32**.

**Scheme 7:**
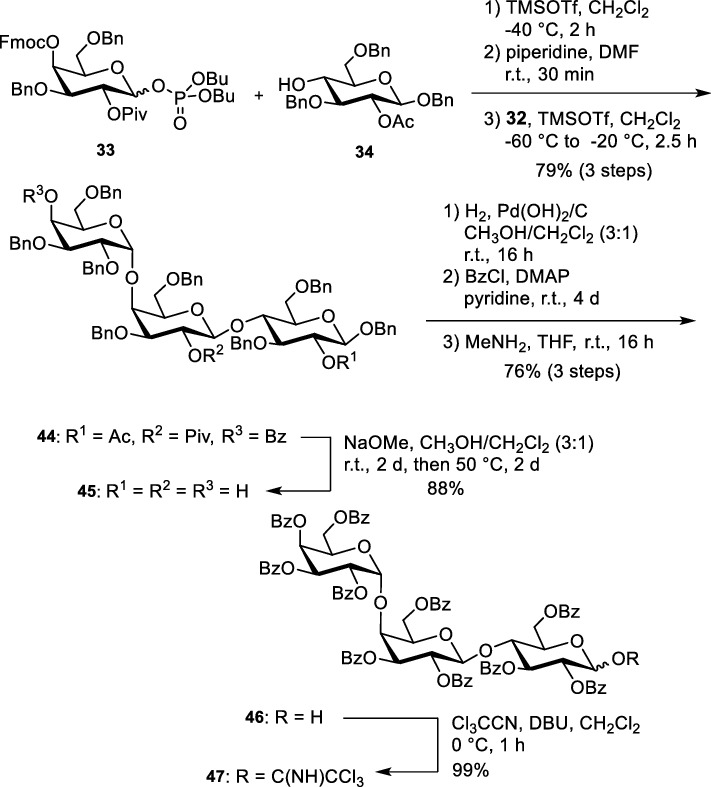
Assembly of the protected globotriaoside head group (Schütte et al. 2014)

With the three monosaccharide building blocks the trisaccharidic carbohydrate head was assembled. The sequence starts with the reaction of buildings blocks **33** and **34**. TMSOTf was used as Lewis acid to promote the cleavage of the phosphate (Scheme 7). Pivaloate as neighbouring participating group ensured the formation of a β-linkage. Subsequent removal of the Fmoc protecting group followed by the second glycosylation with building block **32** resulted in trisaccharide **44** in 76% yield (over three steps). Remote participation of the benzoate in position 4 of the terminal galactose and low temperature (− 60 °C) secured pure α-selectivity. Previous experiments of a completely benzylated galactosyl phosphate revealed a much lower yield and a worse selectivity. The commonly used procedure to cleave all the benzyl protecting groups by hydrogenolysis at the very end of an oligosaccharide assembly is not a choice, because the double bond of the sphingosine would be destroyed during this hydrogenation. Therefore, all the protecting groups of the globotriaose unit were exchanged by benzoate protecting groups before the lipid part is attached. Such benzoates can be easily cleaved at the end of the synthesis under basic conditions before lipid attachment. To get perbenzoylated material, the three different acyl groups were removed to yield **45**, followed by a hydrogenolysis of the benzyl ethers using Pearlman’s catalyst. Perbenzoylation of the naked trisaccharide was performed followed by cleavage of the anomeric benzoate using methyl amine. Hemiacetal **46** was converted into trichloroacetimidate **47** in quantitative yield.

**Scheme 8:**
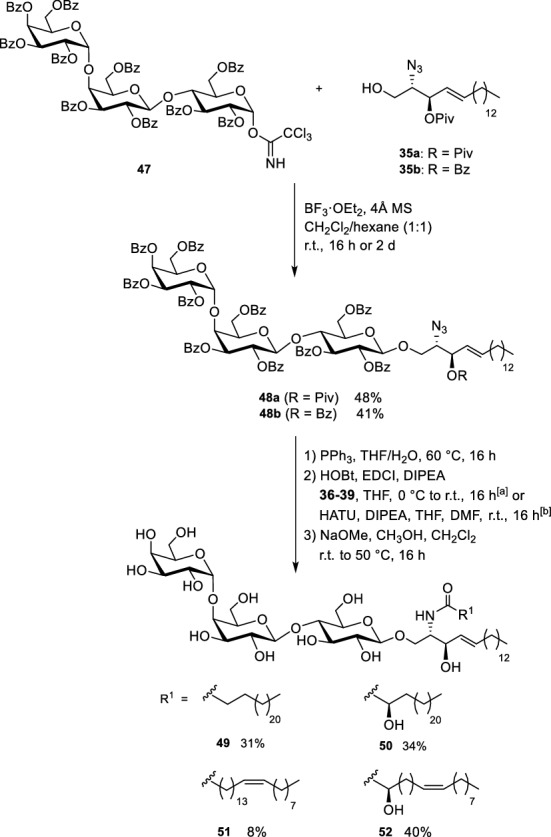
Union of the lipophilic part with the carbohydrate head group, amide formation and global deprotection (Schütte et al. 2014, 2015)

With the protected trisaccharide acceptor **47** in hand, the non-labelled Gb_3_ sphingolipids can be synthesized by the union with the lipophilic sphingosine alcohol **35a**/**35b**. BF_3_⋅OEt_2_ was used as catalyst to trigger this difficult glycosylation reaction; moderate yields were obtained (Scheme 8).

**Fig. 3:**
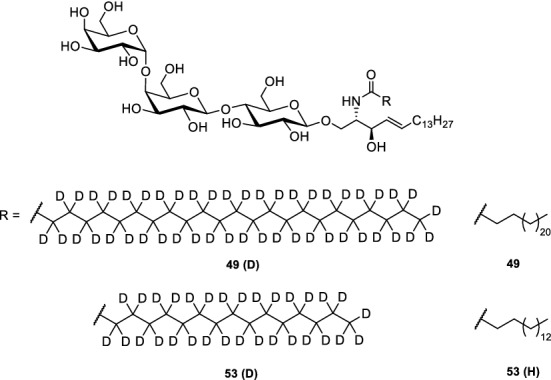
Deuterated Gb_3_ derivative **49(D)** and **53(D)** (Bosse et al. 2019)

Reduction of the azide moiety by Staudinger reaction and direct coupling with the fatty acids **36–39** under common peptide coupling conditions (e.g., HATU or HOBt/EDCI) afforded four protected glycosphingolipids with different fatty acid chains. Global deprotection was performed under Zemplén conditions resulting in the glycosphingolipids **49–52**.This modular procedure readily allowed us to also synthesize Gb_3_ derivatives with deuterated fatty acids (Bosse et al. 2019). Here, commercially available completely deuterated fatty acids were used for the amidation procedure (Fig. 3).

**Fig. 4:**
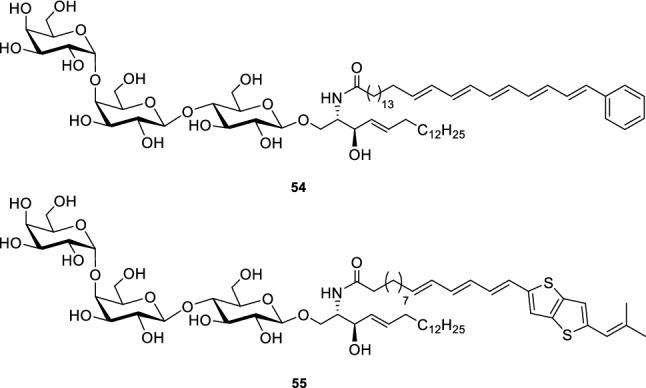
Fatty acid labelled Gb_3_ derivatives **54** and **55** (Patalag et al. 2017)

To generate the fatty acid labelled Gb_3_ derivatives (Fig. 4), the same protocol was pursued making use of the fatty acids **25** and **31** (cp. Schemes 3 and 4) (Patalag et al. 2017).

For the head group labelled Gb_3_ derivatives, a fluorescent dye was attached to the carbohydrate head group via an oligoethylene glycol (OEG) linker. However, following this strategy includes that the attached fluorophore must not alter the binding behaviour of Shiga toxin B subunits (STxB). Hence, we chose to attach the fluorophore via an OEG spacer to the 2′-OH group of the middle galactose Gb_3_, known to not participate in STxB binding (Kitov et al. 2003; Kitov and Bundle 2001; Ling et al. 1998). This synthetic route has the advantage that the influence of the fatty acid on the partition of Gb_3_ in phase-separated bilayers can be readily investigated.

**Scheme 9:**
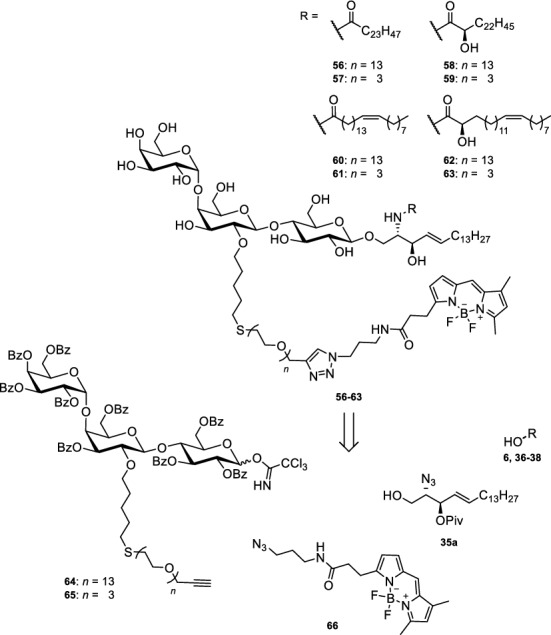
Retrosynthetic analysis of Gb_3_ derivatives with fluorophore attached to the carbohydrate head group via an OEG linker (Sibold et al. 2019)

The major goal was to design a modular convergent synthesis for head group labelled Gb_3_ derivatives with different fatty acids and different OEG linker lengths allowing us to vary the fatty acid and the fluorophore with minimal synthetic effort (Scheme 9). In contrast to semisynthetic methods, this approach ensures that a highly defined material is obtained being prerequisite for quantitative biophysical investigations. The retrosynthetic analysis of the desired structures **56–63** led to four different components. The BODIPY dye **66**, which is commercially available was planned to be attached to the carbohydrate head group in the last step of the synthesis via a Huisgen (3 + 2)-cycloaddition (which is often also termed as click-chemistry). As mentioned before, we anticipated that the sphingosine core should be introduced as azido sphingosine **35a** in which the azide serves as a masked amine. Amidation with four different fatty acids would lead to the respective glycolipids of a C_24_ chain length.

The trisaccharide building block is much more difficult to access than the previously shown one without the OEG linker. To attach the OEG at the 2-hydroxyl group of the middle galactose a different building block for this monosaccharide had to be designed. Instead of the permanent pivaloyl ester (in Scheme 7) we used Fmoc as easily cleavable temporary protecting group (Oberli et al. 2008; Werz and Seeberger 2005) in position 2 and PMB in position 4.

Fmoc in **68** ensures as neighbouring participating group the β-selectivity, whereas PMB is easily cleavable in the presence of acids.

**Scheme 10:**
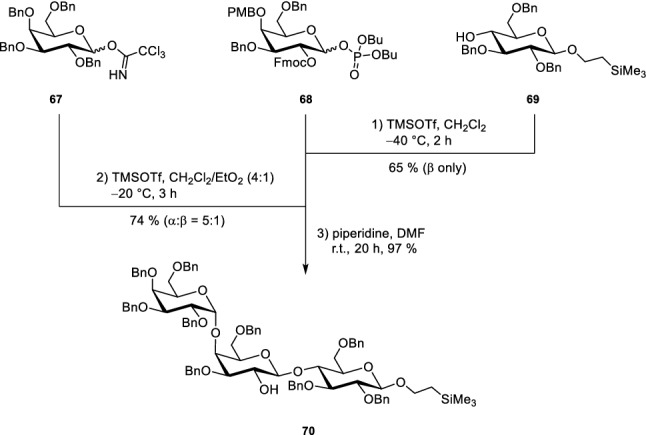
Trisaccharide assembly with a free 2-hydroxyl group of the second galactose unit (Sibold et al. 2019)

**Scheme 11:**
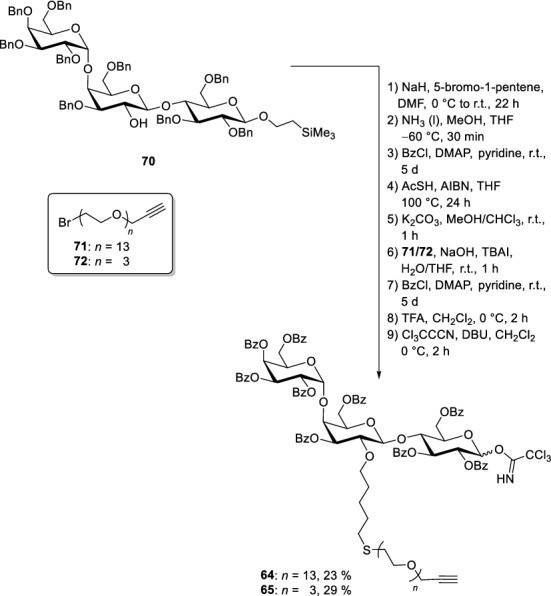
Exchange of benzyl protecting groups to benzoates and attachment of the OEG linker (Sibold et al. 2019)

Indeed, the assembly showed that already the presence of the Lewis acidic TMSOTf proved to be sufficient to cleave PMB after the formation of the disaccharide (Scheme 10). Glycosylation with the third monosaccharide building block **67** afforded the core trisaccharide. Piperidine as amine base led to a deprotection of the Fmoc and the generation of **70**. Trisaccharide **70** was then equipped with a pentenyl chain in the position, where the flexible OEG linker connected to the fluorophore needs to be attached (Scheme 11). In the subsequent step all benzyl protecting groups were removed under Birch conditions. During this procedure the anomeric CH_2_CH_2_TMS group and the double bond in the pentenyl handle stayed intact. All emerging hydroxyl groups of the trisaccharide were converted into benzoates. Such esters are advantageous as they can be easily removed at the end of the synthesis without affecting the double bond in the lipid part.

**Scheme 12:**
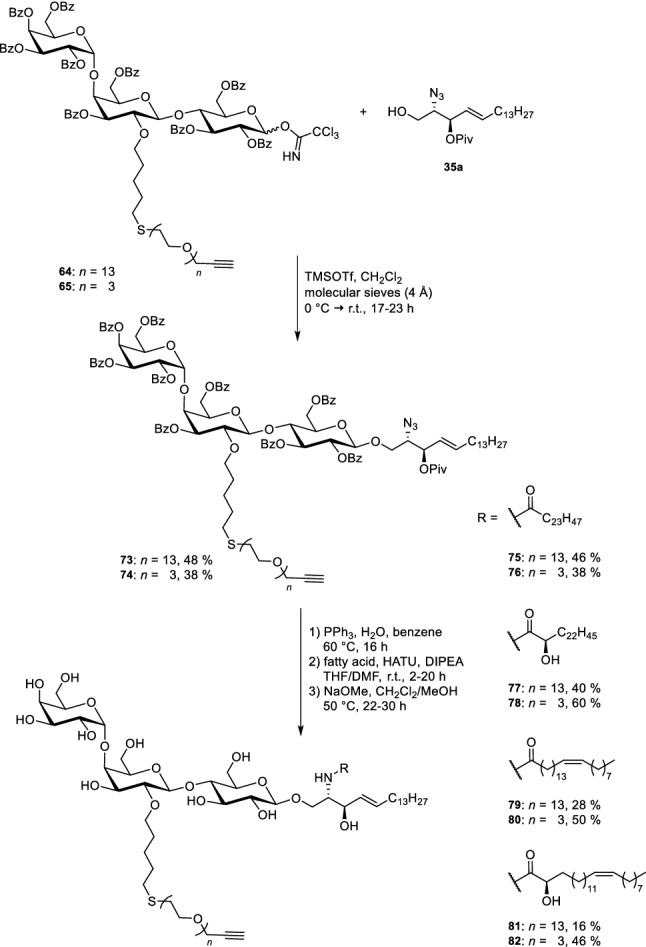
Union of carbohydrate head group with the lipophilic parts (Sibold et al. 2019)

In a further step, the OEG linker is attached to the double bond. To achieve this, a thiol was first introduced as a linking element by a radical approach. The highly nucleophilic thiolate easily reacted with OEG bromides **71** (13 ethylene glycol units) and **72** (3 ethylene glycol units), respectively. To ensure a full protection of all hydroxyl groups, the benzoylation step had to be repeated. Finally, the silylethyl group at the anomeric position was removed with trifluoroacetic acid and the emerging hemiacetals were converted into the corresponding trichloroacetimidates **64** and **65**. To build up the glycolipid, the trichloroacetimidates **64**/**65** were reacted with protected azidosphingosine **35a**, which was prepared from the naturally occurring amino acid l-serine. TMSOTf served as Lewis acid for this glycosylation reaction and afforded compounds **73** and **74** in moderate yields (Scheme 12). The azide was transformed in a Staudinger reduction to an amine which was coupled directly to the respective fatty acids; OEG-modified glycosphingolipids were obtained. Global deprotection was achieved using sodium methoxide as base furnishing compounds **75–82**, respectively. Click chemistry—or in other words a Cu-catalysed Huisgen (3 + 2)-cycloaddition reaction (Breugst and Reissig 2020)—was used to attach the commercially available BODIPY dye **66** to the terminal triple bond of the linker.

**Scheme 13:**
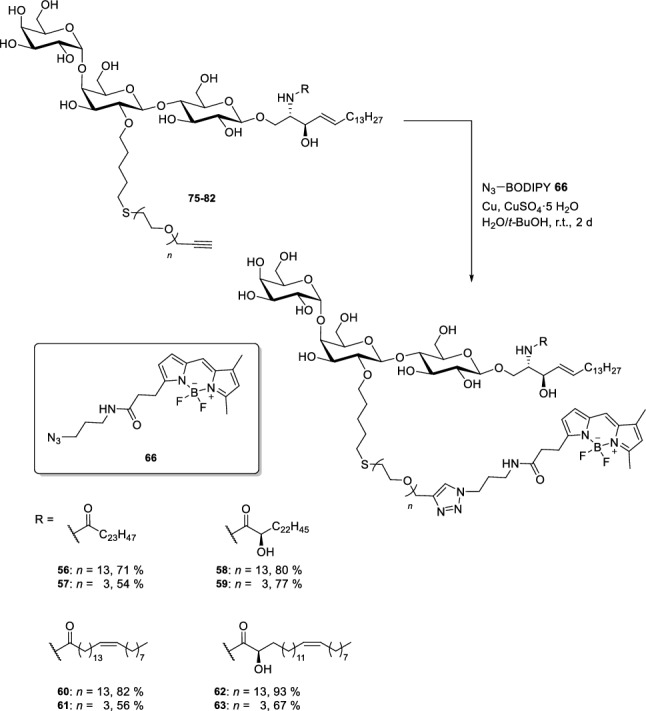
Click chemistry for the synthesis of eight fluorescent Gb_3_ glycosphingolipids (Sibold et al. 2019)

Eight different fluorescently labelled glycosphingolipids **56–63**, varying in the OEG linker length and the acyl chain of the fatty acid were obtained (Scheme 13).

## Analysis of STxB binding affinities as a function of different Gb_3_ glycosphingolipids

The functionality of the synthesized Gb_3_ species can be verified by their ability that STxB binds specifically to membranes doped with the receptor lipids. To quantitatively determine the binding affinity, adsorption isotherms of STxB interacting with Gb_3_-doped monolayers were measured by means of surface plasmon resonance (SPR) technique and the dissociation constants were determined for the non-fluorescently labelled Gb_3_ species. According to the procedure described by Nakajima et al. (Nakajima et al. 2001), vesicles composed of DOPC/cholesterol (Chol)/Gb_3_ were spread on an octanethiol functionalized gold sensor chip. After the monolayer has been established, different STxB concentrations were added and the change in reflectivity was read out as a function of protein concentration. For all synthesized non-labelled Gb_3_ species, a specific binding of STxB with affinities in the nanomolar regime was measured (Table 1).

**Table 1:** Dissociation constants obtained by SPR measurements (Bosse et al. 2019; Schütte et al. 2015)

Lipid composition	*K*_D_/nM
DOPC/Gb_3porc_ (95:5)	3 ± 2/5 ± 1
DOPC/**49** (Gb_3_C_24:0_) (95:5)	4 ± 1
DOPC/**50** (Gb_3_C_24:0 2-(*R*)-OH_) (95:5)	7 ± 2
DOPC/**51** (Gb_3_C_24:1_) (95:5)	12 ± 1
DOPC/**52** (Gb_3_C_24:1 2-(*R*)-OH_) (95:5)	8 ± 2
DOPC/Chol/**52** (75:20:5)	14 ± 3

STxB (pentamer) was bound to Gb_3_ doped lipid monolayers. Fatty acid composition of Gb_3porc_: C_16:0_ (21%), C_18:0_ (4%), C_18:1_ (2%), C_22:0_ (14%), C_23:0_ (1%), C_24:0_ (19%), C_24:1_ (11%), C_22:0 2-(*R*)-OH_ (4%), C_24:0 2-(*R*)-OH_ (5%) and C_24:1 2-(*R*)-OH_ (17%)

Only small trends concerning the binding affinities were found. While the highest affinity was determined for the Gb_3_ mixture from porcine erythrocytes with a *K*_D_ of about 4 nM identical to that determined for Gb_3_C_24:0_ and very similar to that for the hydroxylated species, the monolayers containing the unsaturated Gb_3_ species show slightly higher *K*_D_ values. In previous reports, binding studies were only performed with Gb_3_ species harbouring fatty acids of different length (Binnington et al. 2002; Kiarash et al. 1994; Mahfoud et al. 2009). As the fatty acid chain length in this study is identical for all Gb_3_ species, we can conclude that the unsaturation is responsible for the different binding affinity probably owing to an altered packing property of the Gb_3_ molecules in the membrane. Similarly, the binding affinity is slightly diminished if Chol is added to the mixture. To illuminate the aspect of membrane organisation in more detail, we performed fluorescence and atomic force microscopy imaging on lipid bilayers resembling the outer leaflet of the plasma membrane of eukaryotic cells (van Meer and Kroon 2011), doped with the different Gb_3_ sphingolipids.

## Phase behaviour of liquid ordered/liquid disordered coexisting supported membranes doped with different Gb_3_ sphingolipids

**Fig. 5:**
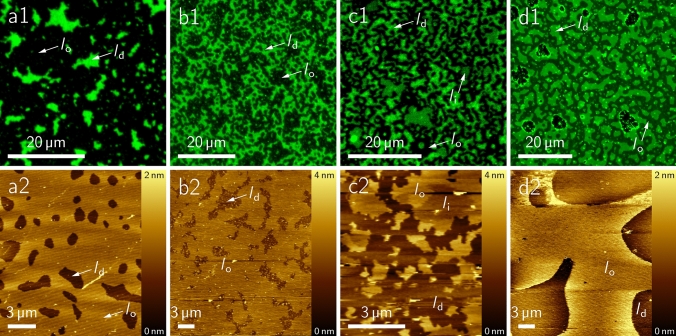
Solid supported membranes composed of DOPC/SM_porc_/Chol/Gb_3_ (39.5:35:20:5) harbouring the different non-labelled Gb_3_ sphingolipids and doped with a *l*_d_ phase marker (0.5 mol%). The top row shows the fluorescence micrographs. The *l*_d_ phase is visualized in green. The bottom row shows the corresponding AFM topography images. **a** **49** (Gb_3_C_24:0_), **b** **50** (Gb_3_C_24:0 2-(*R*)-OH_), **c** **51** (Gb_3_C_24:1_) and **d** **52** (Gb_3_C_24:1 2-(*R*)-OH_). Figure with permission adapted from (Schütte et al. 2014, 2015)

The outer leaflet of the plasma membrane of eukaryotic animal cells contains sphingomyelin (SM) and Chol. A general mixture frequently used in literature, which is also known as the “raft mixture” is composed of DOPC/SM/Chol (40:40:20). At room temperature, this mixture phase separates into a liquid ordered (*l*_o_) and a liquid disordered (*l*_d_) phase. We used mixtures composed of DOPC/SM_porc_/Chol/Gb_3_ (40:25:20:5), where 5 mol% of SM from porcine brain (SM_porc_) was replaced by Gb_3_ (Schütte et al. 2014, 2015). As expected, all lipid mixtures show *l*_o_/*l*_d_ coexistence. The two different phases can be distinguished by the lipid fluorophore that preferentially partitions into the *l*_d_ phase (Sezgin et al. 2012) and the height difference of about 0.6–0.8 nm between *l*_o_ and *l*_d_ phase as obtained by atomic force micrographs (Azouz et al. 2019; Sullan et al. 2010) (Figs. 5, 7). Of note, in case of Gb_3_C_24:1_, three different phases could be distinguished in the fluorescence micrographs, which we assigned to the *l*_o_ phase (black), an intermediate phase (*l*_i_ phase, dark green) and the *l*_d_ phase (green) (Fig. 5c). The three different phases were also reflected in different heights of the phases in the AFM topographs with a larger height difference of 1.6 ± 0.2 nm between the lowest and the highest phase compared to the other cases (Fig. 7). The images clearly show that the fatty acid attached to Gb_3_ significantly influences the phase behaviour of the DOPC/SM_porc_/Chol lipid mixtures even though it only constitutes 5 mol% of the total lipids. The result suggests that Gb_3_ strongly interacts with other lipids and probably partitions between the *l*_o_ and *l*_d_ phase differently, dependent on the fatty acid composition. Indeed, this is what we found using fluorescently labelled Gb_3_ sphingolipids. This notion is also reflected in the differences in the *l*_o_ fractions. Even though the overall lipid composition is the same, the *l*_o_ fraction in case of the hydroxylated species (**50**) is significantly lower (56 ± 5%) compared to the saturated non-hydroxylated one (**49**, 74 ± 7%) (Fig. 7).

**Fig. 6:**
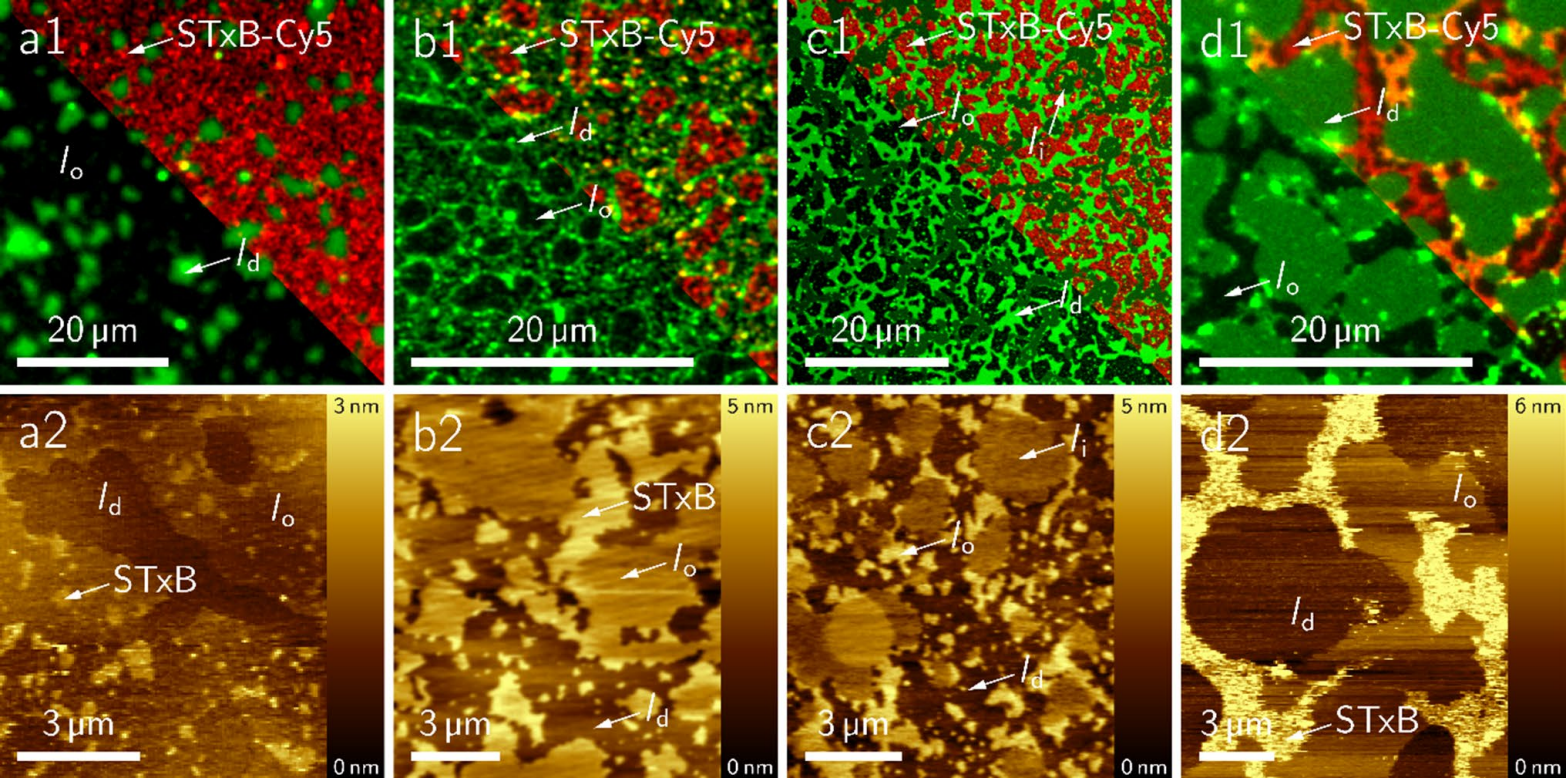
Solid supported membranes composed of DOPC/SM_porc_/Chol/Gb_3_ (39.5:35:20:5) harbouring the different non-labelled Gb_3_ sphingolipids and doped with a *l*_d_ phase marker (0.5 mol%) after incubation with 60 nM STxB-Cy3 (pentamer). The top row shows the fluorescence micrographs. The *l*_d_ phase is visualized in green, the protein in red (overlay shown in the right part of the images). The bottom row shows the corresponding AFM topography images. **a** **49**, **b** **50**, **c** **51**, and **d** **52**. Figure with permission adapted from (Schütte et al. 2014, 2015)

**Fig. 7:**
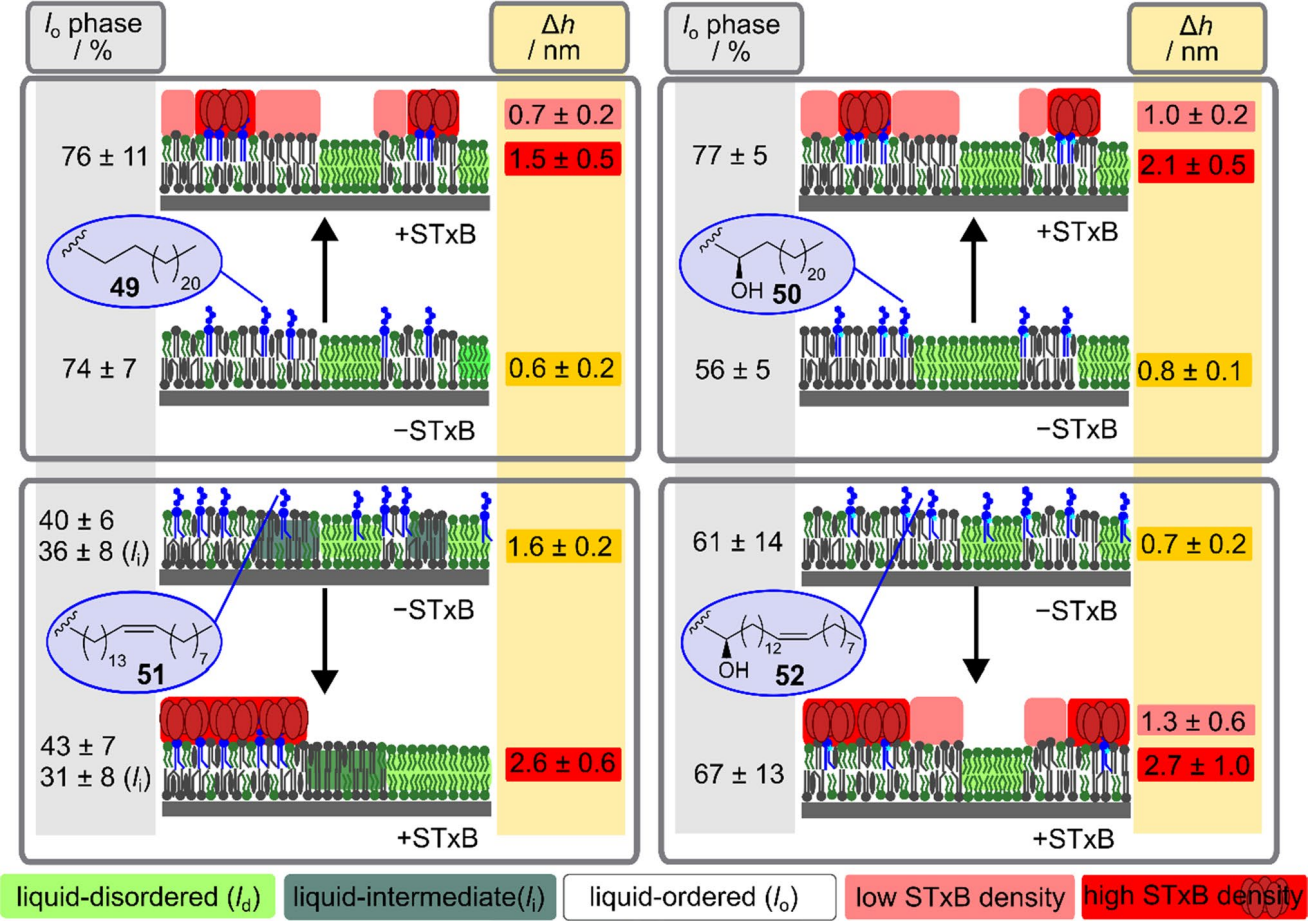
Schematic drawing of the phase behaviour of phase-separated lipid membranes (DOPC/SM_porc_/Chol/Gb_3_, 40:35:20:5) containing different Gb_3_ species in absence (− STxB) and presence (+ STxB) of STxB. The different lipid phases observed in the fluorescence images are colour-coded as given in the legend below. Each grey box reports the results obtained with one of the four different Gb_3_ species (shown in the blue bubble). The fraction of the *l*_o_ phase area as obtained from the fluorescence images is given as *l*_o_ phase/ %. The height differences determined from the AFM topography images are given for the difference between *l*_o_ and *l*_d_ phase (yellow), the difference between *l*_d_ phase and areas of low STxB density (light red) and between *l*_d_ phase and areas of high STxB density (red). The localization of the Gb_3_ sphingolipids cannot be determined from these experiments. However, further experiments on GUVs with fluorescently labelled Gb_3_s will shine more light on this aspect

The question next arises how does binding of STxB alters the phase behaviour of the coexisting supported lipid bilayers. It has been shown previously, that binding of STxB to Gb_3_ alters the lipid membrane organization (Römer et al. 2007; Safouane et al. 2010). The protein can bind up to 15 receptor molecules suggesting a mutual influence of the membrane organisation on the bound protein and vice versa. Hence, it is well conceivable that the difference in partitioning of the Gb_3_ sphingolipids is also reflected in a different reorganisation behaviour of the membranes upon STxB binding. To investigate this aspect in more detail, fluorescently labelled STxB (60 nM STxB-Cy3) was added to the phase-separated solid supported bilayers and fluorescence and atomic force microscopy images were taken (Fig. 6). From previous studies, it is known that STxB binds exclusively to the *l*_o_ phase implying that Gb_3_ is localised in the *l*_o_ phase after protein binding (Windschiegl et al. 2009). Indeed, also in case of the chemically well-defined Gb_3_ molecules, STxB binds solely to the *l*_o_ phase. Even in the case of the unsaturated Gb_3_ (**51**), STxB binds only to the *l*_o_ phase (dark), while the *l*_i_ phase (dark green) is protein free. However, the mode of protein binding appears to be different. Given by the observed height differences in the atomic force micrographs, we distinguish between STxB clusters (high STxB density) indicated by a height difference of Δ*h* between 1.5–2.5 nm (refenced to the *l*_d_ phase) and less densely packed STxB with only a height difference of Δ*h* = 0.7–1.3 nm as observed by AFM topographs (Fig. 7).

STxB binding to the different Gb_3_ species, except for the unsaturated one (**51**), results in the formation of high-density protein clusters and low-density protein areas. Such clusters in combination with Gb_3_ molecules harbouring a saturated fatty acid might inhibit membrane bending (Römer et al. 2007) due to the rigid structure (Pezeshkian et al. 2015; Watkins et al. 2014). The unsaturated Gb_3_ species **51** appears to be the most important one for the first step of Shiga toxin internalisation. STxB binding leads only to protein clusters concomitant with a large area demand of the lipids (Gracià et al. 2010; Kollmitzer et al. 2013). This is supposed to induce negative curvature eventually leading to the observed Shiga toxin induced invaginations. Another aspect is the reorganisation of the lipids between *l*_o_ and *l*_d_ phase. Here, membranes containing Gb_3_ molecules with an α-hydroxylation show an increase in the *l*_o_ fraction after protein binding suggesting a large redistribution of *l*_o_ phase lipids.

These results let us conclude that the Gb_3_ distribution between *l*_o_ and *l*_d_ phase might be different dependent on the fatty acid. However, while we can safely assume that the majority of Gb_3_ molecules are localised, where STxB has been bound (Schütte et al. 2014, 2015), it remains unclear how the Gb_3_ species are distributed in *l*_o_/*l*_d_ coexisting membranes.

## Localizing fluorescently labelled Gb_3_ glycosphingolipids in lipid membranes of GUVs

To be able to address the question, where Gb_3_ is localized prior to STxB binding, we pursued an approach of fluorescently labelling Gb_3_. In our first strategy, we synthesized Gb_3_ molecules with labelled fatty acids (Fig. 4). However, as it turned out that the binding behaviour of STxB was altered in the presence of these molecules, a second labelling strategy was followed, in which a fluorophore was attached to the head group of Gb_3_ (Scheme 13).

**Fig. 8:**
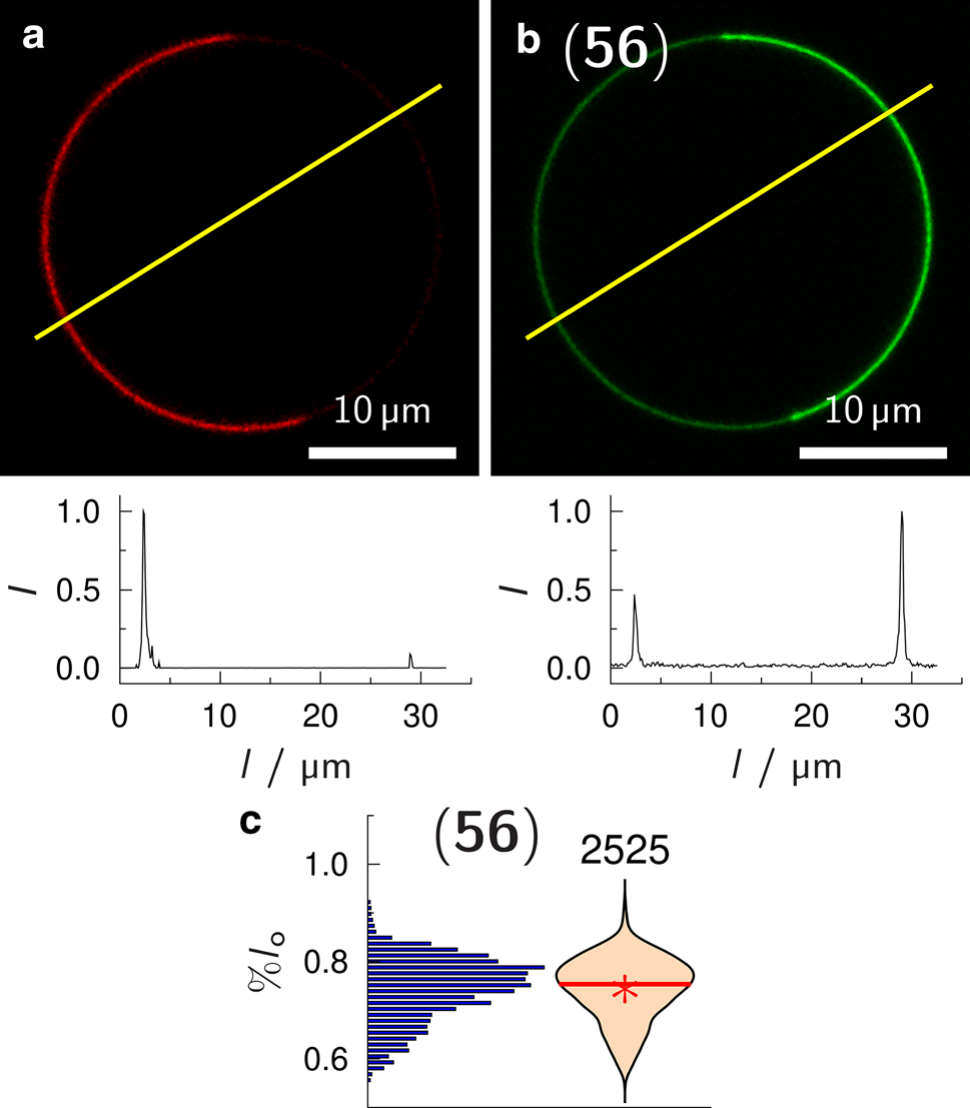
Confocal fluorescence images of a phase-separated GUV composed of DOPC/SM_porc_/Chol/**56**/Dy731-DOPE (39/39/20/1/1). **a** Dy731-DOPE fluorescence (red). **b** **56** fluorescence (green). The yellow lines show the position, where the fluorescence intensity profiles (bottom images) were taken. These intensity profiles were used to calculate the apparent partition coefficient %*l*_o_ = 68.2%. **c** Histogram and corresponding violin plot obtained from 60 GUVs (2525 intensity profiles) with the composition as in (**a**/**b**). The red solid line shows the median, the red star the mean value. Figure with permission adapted from (Sibold et al. 2019)

Two different fatty acids with either a phenylpentaene moiety (Scheme 3) or a thienothienyl-moiety (Scheme 4) (Patalag et al. 2017) were attached to obtain the Gb_3_ species **54** and **55**. To analyse the partition of these Gb_3_ sphingolipids in phase-separated membranes, we made use of giant unilamellar vesicles (GUVs). GUVs can be doped with lipid-like dyes that partition specifically either in the *l*_o_ or *l*_d_ phase (Baumgart et al. 2007; Sezgin et al. 2012). GUVs were prepared by the electroformation technique and were composed of DOPC/SM/Chol/Gb_3_/Dy731-DOPE (39.5/35/20/5/0.5). Importantly, we have chosen a *l*_d_ phase marker (Dy731-DOPE) with an absorption spectrum shifted to long wavelengths (*λ*_ex_ = 732 nm) to not overlap with the emission spectra of the Gb_3_ molecules, which would result in an undesired fluorescence resonance energy transfer. Dy731-DOPE was chemically synthesized and partitions into the *l*_d_ phase (Patalag et al. 2017). To determine the fraction of Gb_3_ species partitioning into the *l*_o_ phase, we read out the fluorescence intensities of the *l*_o_ phase and the *l*_d_ phase from the intensity line profiles of confocal images (Fig. 8), namely, *I*_*l*o_ and *I*_*l*d_. According to Eq. 1 an apparent partitioning coefficient (%*l*_o_) was calculated:

1 $$\mathrm{\%}{l}_{\mathrm{o}}=\frac{{I}_{{l}_{\mathrm{o}}} \bullet 100\mathrm{\%}}{{I}_{{l}_{\mathrm{o}}}+{I}_{{l}_{\mathrm{d}}}},$$

**Fig. 9:**
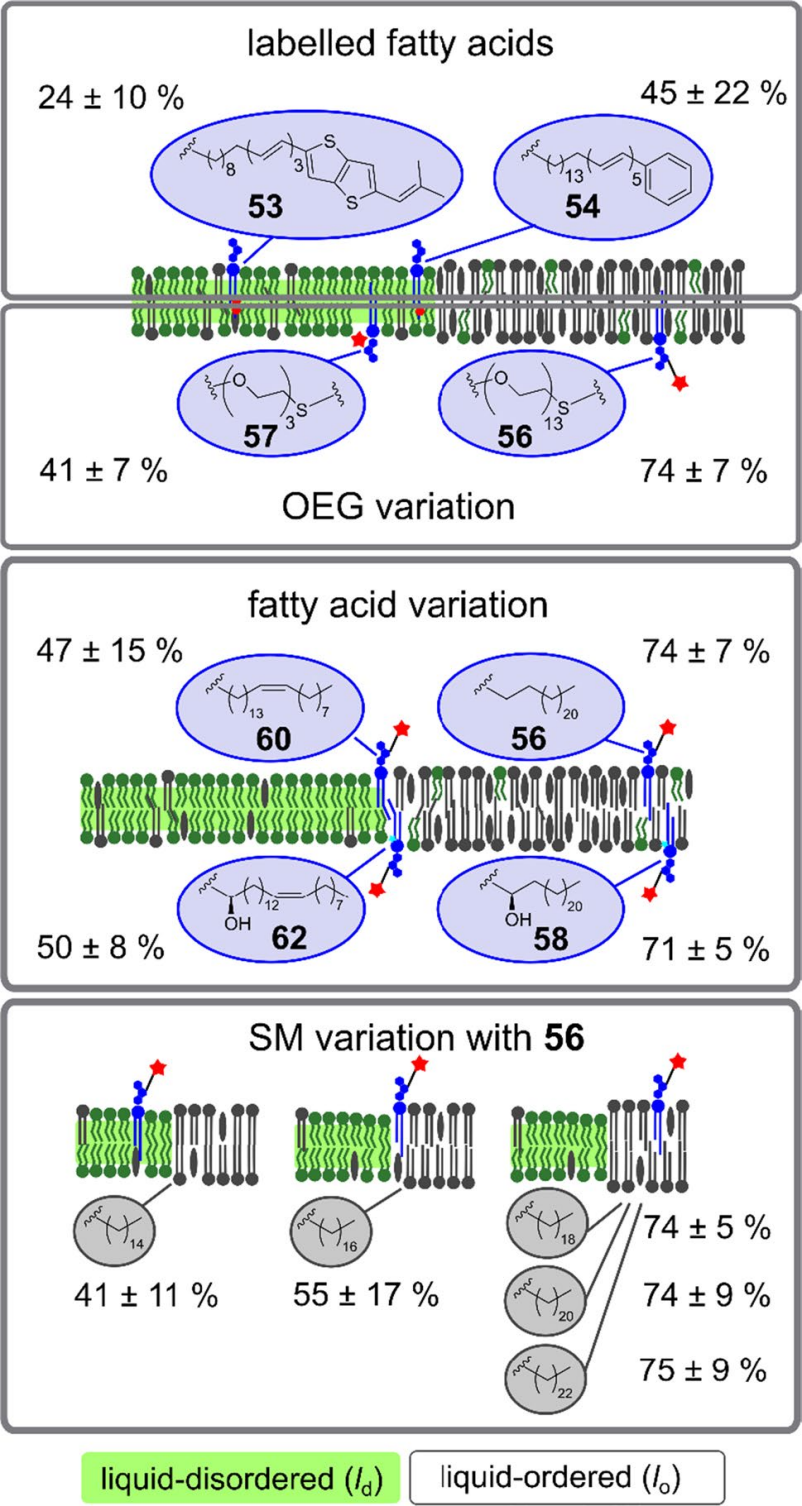
Schematic drawing reporting the distribution of the fluorescently labelled Gb_3_ sphingolipids (blue bubbles) in *l*_o_/*l*_d_ phase-separated membranes obtained from fluorescence images of GUVs with the corresponding apparent partition coefficients %*l*_o_ (Eq. 1). The first grey box shows the results obtained for the fatty acid labelled Gb_3_ sphingolipids, the second box shows the two head group labelled Gb_3_s with OEG linkers differing in their length and a C_24:0_ fatty acid attached. The third box summarizes the results obtained for the Gb_3_ sphingolipids with the long OEG linker and varying fatty acids. The fourth box illustrates the distribution of the Gb_3_ with the C_24:0_ fatty acid attached and the long OEG linker in presence of sphingomyelin varying in their fatty acid length as shown in the grey bubbles

For compound **54**, %*l*_o_ was determined to be 45%, whereas %*l*_o_ = 24% was calculated for compound **55** (Fig. 9). However, even though we could show that the Gb_3_ species are capable of binding STxB using fluorescently labelled Cy3-STxB, the results also demonstrated that STxB binds to the *l*_d_ phase in contrast to what is known from naturally occurring Gb_3_-containing membranes.

This finding clearly demonstrates that small changes in the packing density in the *l*_o_ phase induced by bulky groups such as the phenyl ring and the thiophene ring can largely alter the phase behaviour of the glycosphingolipids. It has been reported previously that sphingolipids containing BODIPY-FL or NBD in the fatty acid chain do not partition in the *l*_o_ phase owing to the bulkiness inserted into the hydrophobic part of the membrane (Baumgart et al. 2007; Komura et al. 2016; Sengupta et al. 2008; Sezgin et al. 2012). Similarly, it was observed that G_M1_ being the natural receptor lipid of cholera toxin, partitions and binds cholera toxin exclusively in the *l*_d_ phase, if the acyl chain is labelled with BODIPY-FL, even though it is known that cholera toxin binds to the *l*_o_ phase in case of unlabelled G_M1_ (Bacia et al. 2005; Komura et al. 2016; Sezgin et al. 2012).

To not touch the fatty acid, another strategy was pursued based on the modification of the head group of Gb_3_ (Scheme 13). As it is well conceivable that even a fluorophore attached to the head group still influences the phase behaviour of *l*_o_/*l*_d_ phase-separated bilayers, we synthesized two Gb_3_ series, one with three OEG units, the other one with 13 OEG units. Indeed, the length of the spacer influences the partition of the Gb_3_ species. The Gb_3_ sphingolipids with the longer linker (13 OEG units) partitions by 15–33% more in the *l*_o_ phase than those with only 3 OEG units (Fig. 9). Previous studies reported on similar observations. A lipid that is known to prefer the *l*_o_ phase was found in the *l*_d_ phase, if the fluorophore was directly coupled to the head group (Klymchenko and Kreder 2014). If a linker was placed between the fluorophore and the head group, the lipid re-partitions to the *l*_o_ phase (Honigmann et al. 2013). Apparently, a long linker helps separating the fluorophore from the membrane interface so that it does not disturb the lipid packing. Lipids with longer linkers partition more likely in the *l*_o_ phase (Bordovsky et al. 2016; Momin et al. 2015).

As we assume that the fluorescently labelled Gb_3_ with the long linker reflects the natural behaviour of the Gb_3_ sphingolipids in a better way, we focused our study on these species and analysed the influence of the fatty acid on the partition behaviour. As expected, Gb_3_ with a saturated fatty acid partitions predominantly in the *l*_o_ phase (Fig. 9) (74% for **56** and 71% for **57**). If the saturated fatty acid is replaced by a mono-unsaturated fatty acid, an almost equal partitioning of Gb_3_ between *l*_o_ and *l*_d_ phase is found (47% for **60** and 50% for **61**). The influence of an unsaturated fatty acid on the phase behaviour of coexisting lipid membranes has been also described by Maté et al. (Maté et al. 2014). They reported that if the fatty acid of SM is changed from a C_24:0_ fatty acid to a C_24:1_ fatty acid in a DOPC/SM/Chol membrane, the phase separation disappears. These findings support our notion that the packing of the unsaturated Gb_3_ species disfavours its partition in the *l*_o_ phase, similarly to what has been reported by Legros et al. for in vivo conditions (Legros et al. 2017). For primary human blood brain barrier endothelial cells, they found that Gb_3_ with a C_24:1_ fatty acid resides more strongly in non-detergent-resistant membranes compared to Gb_3_ with a C_24:0_ fatty acid. Our results have also implications for the proposed membrane reorganisation upon STxB binding. As more Gb_3_ is localized in the *l*_d_ phase prior protein binding, a larger reorganisation will take place upon STxB binding assuming that the majority of Gb_3_ is in the *l*_o_ phase after STxB has been bound.

Besides the saturation of the fatty acid, we also investigated the influence of the α-hydroxylation on the Gb_3_ partition behaviour. Our results show that the OH-group does not influence the Gb_3_ phase distribution. Others also found that the introduction of an OH-group does not alter the order parameter of the lipids (Morrow et al. 1995a, b). However, we have seen that in case of supported membranes, those lipid bilayers with α-hydroxylated Gb_3_ sphingolipids exhibit a reduced *l*_o_ phase fraction (Schütte et al. 2014). Slotte (2013) showed that the 2-OH group increases the hydration in the membrane interface. Moreover, it decreases the affinity of a sphingolipid for sterols, which means that less Chol is recruited into the *l*_o_ phase in case of hydroxylated Gb_3_ compared to the non-hydroxylated species. This results in a smaller *l*_o_ fraction, even though the amount of Gb_3_ in the *l*_o_ fraction is the same.

We not only investigated the influence of the fatty acid of the Gb_3_ molecules, but also looked into more detail into the impact of the fatty acid of SM. In general, long chain sphingolipids are known to be able to interdigitate. For SM with fatty acids of more than 20 carbon atoms, it was reported that they even interdigitate in the liquid-crystalline phase (Maulik et al. 1986; Niemelä et al. 2006; Takahashi et al. 2007). Glycosphingolipids with a fatty acid of 24 carbon atoms can also interdigitate (Niemelä et al. 2006; Róg et al. 2016). Thus, we hypothesized that the SM species influence the partitioning of the Gb_3_ molecules by a specific and probably similar packing behaviour. In a series of experiments with SM with different fatty acids ranging from C_16:0_ to C_24:0_, we saw a clear trend that the partition of Gb_3_ into the *l*_o_ phase was favoured for SM harbouring fatty acids with 18 or more carbon atoms, which might indicate that the packing into the *l*_o_ phase is favoured if the chain lengths match with the possibility to interdigitate (Fig. 9).

The ultimate question remained whether STxB is capable of binding to the head group fluorescently labelled Gb_3_ molecules. Indeed, we could demonstrate that STxB binds to **56** and that it is localized in the *l*_o_ phase as expected from the natural Gb_3_ molecules. However, to readily visualize STxB binding, 5 mol% of Gb_3_ were required. This concentration already leads to partial self-quenching of the fluorescence which prevented us from quantifying the Gb_3_ distribution after protein binding.

## Packing behaviour of Gb_3_ glycosphingolipids analysed by NMR spectroscopy

As the packing behaviour of Gb_3_ appears to be an important aspect of its partitioning, we illuminated this aspect using ^2^H solid-state NMR spectroscopy (Bosse et al. 2019). To perform these experiments, perdeuterated Gb_3_ lipid acyl chains were synthesized (Fig. 4). With these compounds in hand, we were able to study the influence of Gb_3_ lipids with a C_24_ fatty acid [**49, 49(D)**] on the properties of phospholipid membranes composed of a *l*_o_/*l*_d_ phase-separated lipid membranes composed of 1-palmitoyl-2-oleoyl-*sn*-glycero-3-phosphocholine (POPC)/N-palmitoyl-D-erythro-sphingosylphosphorylcholine (SMC_16:0_)/Chol/Gb_3_ (40/35/20/5, *n*/*n*) by means of ^2^H solid-state NMR spectroscopy. From the solid-state ^2^H NMR spectra, a phase separation into a POPC-rich *l*_d_ and a SMC_16:0_/Chol-rich *l*_o_ phase was concluded. In the mixture with **49**, SMC_16:0_ showed higher order parameters as it preferentially co-localizes with Chol in the Chol-rich *l*_o_ phase, while POPC showed a lower order parameter being in the *l*_d_ phase which is depleted of Chol. This result is in agreement with previous ^2^H NMR data on raft-like mixtures (Bartels et al. 2008; Bunge et al. 2008; Veatch et al. 2007).

**49(D)** showed an unusual order parameter profile of the acyl chain, which flattens out for the last ~ 6 methylene segments. For the upper chain segments (C_2_–C_16_) the order parameter profile of **49(D)** shows similar order parameters as known for phospholipids in the liquid crystalline state. Such an odd chain conformation could be an indication for a partial chain interdigitation (Lewis et al. 1994; Morrow et al. 1993) and/or a very fluid midplane region of the membrane as a result of a strong mismatch of the fatty acid chain length of Gb_3_ and SM. Indeed, if the fatty acids are similar, i.e., **53** (Gb_3_C_16:0_) is used, the Gb_3_ is well associated with the SMC_16:0_/Chol-rich *l*_o_ phase.

In the presence of STxB, Gb_3_ preferentially partitioned into the *l*_o_ membrane phase as described before (Windschiegl et al. 2009). Especially the short acyl chain **53(D)** showed very similar chain order parameters as SMC_16:0_. Surprisingly, we observed isotropic contributions in the ^2^H NMR powder spectra for all lipids, which were most pronounced for the Gb_3_ molecules in the presence of STxB. Such isotropic contributions are a result of highly curved membrane structures with a curvature radius of < 10 nm. Curvature induction has been proposed to be part of the mechanism of Shiga toxin internalisation.

## Conclusions

With the aim to elucidate the impact of individual Gb_3_ species on the phase behaviour of lipid membranes and their ability to bind STxB, we designed synthetic strategies to chemically access pure Gb_3_ sphingolipids. We developed a modular and very universal chemical approach applicable also to other glycosphingolipids, which allowed us to produce a number of different Gb_3_ species. These Gb_3_ sphingolipids were reconstituted into phase-separated lipid membranes.

Even though these phase-separated membranes contained only 5 mol% of the non-labelled Gb_3_ sphingolipids, they significantly influenced the overall membrane organisation. This is of particular interest in light of lipid reorganisation and protein domain formation as these aspects are discussed to be important during the first step of the internalisation process of Shiga toxin and Shiga-like toxins giving rise to the infection of cells. Our results obtained from artificial membrane systems clearly suggest that the unsaturated Gb_3_ species are the most important ones for the first step of Shiga toxin internalisation. Binding of STxB results in highly dense protein clusters connected with a large area demand of the lipids This leads to an asymmetric reduction in membrane area, which is proposed to be prerequisite for membrane invaginations induced by Shiga toxin. This process is probably even facilitated as the unsaturated species have a lower bending modulus compared to the saturated ones. To address where Gb_3_ is localized before STxB binding, fluorescently labelled Gb_3_ species were synthesized. It has been turned out that great care has to be taken to ensure that the fluorescent label does not influence the phase behaviour of the Gb_3_ molecules as well as their binding behaviour to STxB. Head group labelled glycosphingolipids eventually enabled us to address the question how the fatty acid of a glycosphingolipid influences its distribution in *l*_o_/*l*_d_ phase-separated membranes. Our results clearly demonstrate that the fatty acid (un)saturation significantly shifts the Gb_3_ molecules from the *l*_o_ phase (C_24:0_) to the *l*_d_ phase (C_24:1_). This is another important aspect with respect to lipid reorganisation as STxB exclusively binds to Gb_3_ in the *l*_o_ phase. Upon STxB binding the amount of redistributed Gb_3_ and probably also other *l*_o_ phase lipids then depend on the fatty acid of Gb_3_. The length match of the fatty acids of SM and Gb_3_ appears to play another decisive role in determining, where the Gb_3_ glycosphingolipids are preferentially localized. Overall, it appears that the combination of the attached fatty acids of SM and Gb_3_ impacts the distribution of the Gb_3_ glycosphingolipids. Hence, a picture emerges in which the overall lipid distribution between the different domains in a lipid membrane, which is influenced by the fatty acid composition of Gb_3_ and the Shiga toxin induced membrane reorganisation partially determines the invagination of the protein into the host cell.
